# Cultivating compassion in medicine: a toolkit for medical students to improve self-kindness and enhance clinical care

**DOI:** 10.1186/s12909-024-05270-z

**Published:** 2024-03-15

**Authors:** Krisha K. Mehta, Shafkat Salam, Austin Hake, Rebecca Jennings, Afra Rahman, Stephen G. Post

**Affiliations:** 1 Department of Medicine, Stony Brook University, Stony Brook, NY USA; 2 Stony Brook University, Renaissance School of Medicine, Stony Brook, NY USA

**Keywords:** Compassion education, Medical education, Medical humanities

## Abstract

**Background:**

Compassionate care lies at the foundation of good patient care and is a quality that patients and providers continue to value in the fast-paced setting of contemporary medicine. Compassion is often discussed superficially in medical school curricula, but the practical aspect of learning this skill is often not taught using a formal framework. In the present work, the authors present an 8-session curriculum with a mindfulness-based approach to compassion that addresses this need. It is hypothesized that students in this curriculum will improve in their levels of compassion based on validated scales.

**Methods:**

The curriculum was delivered to fourth-year medical students at Renaissance School of Medicine at Stony Brook University who had just completed their clerkship year. It was developed as a customizable set of modules that could be delivered in various ways. The students were taught with evidence-based cognitive exercises followed by group discussions and written reflections based on compassion-focused thematic questions. All students completed a pre- and post-Self-Compassion Scale, Compassion Scale, and Toronto Mindfulness Scale. Students in this course were compared with students in different courses about non-clinical topics delivered at the same time. Wilcoxon Signed Rank tests and Mann Whitney U tests were used to assess potential associations between pre- and post-survey responses for the validated scales and subscales.

**Results:**

17 fourth-year medical students completed pre- and post-course tests, 11 participated in the compassion curriculum while 6 participated from the other courses. Before any of the courses began, all students performed similarly on the pre-test across all scales. The students in the compassion curriculum demonstrated a significant increase in their total Self-Compassion score by 8.7 [95% CI 4.3 to 13.2] points (*p* = 0.008), total Compassion score by 6.0 [95% CI 1.4 to 10.6] points (*p* = 0.012), and the curiosity component of the Toronto Mindfulness Scale by 4.4 [95% CI 1.0 to 7.7] points (*p* = 0.012). There was no statistically significant difference between pre- and post-tests among the non-compassion curriculum students in the aforementioned scales (*p* = 0.461, *p* = 0.144, *p* = 0.785, respectively).

**Conclusions:**

Our results indicate that the students in our course developed an enhanced ability to engage in self-compassion, to understand the shared human experience, and to be motivated to act to alleviate suffering. Regardless of a program’s existing compassion education, this customizable model allows for easy integration into a medical student’s crowded curriculum. Furthermore, although teaching compassion early and often in a clinician’s training is desirable, our study that targeted fourth-year medical students suggests an additional benefit of rekindling the loss of compassion well described in a medical student’s clinical years.

## Introduction

Compassion is defined as experiencing an authentic desire to help another person secondary to an emotional response to that person’s pain or suffering. Not only is compassion about feeling the desire, but also in enacting such a will. Although it seems unquestionable that compassionate care lies at the foundation of medicine and should be employed with all patients, there remains an obvious deficit in applying compassionate care in clinical medicine [1]. A 2010 national survey of 800 hospitalized patients and 510 physicians showed that nearly half of the patients and physicians felt that compassionate care is missing from the current healthcare system, signaling an incredibly urgent “compassion crisis” [2].

A consensus on nomenclature is required to advance our understanding and eventual education of this subject. Empathy involves two stages: the affective stage is the sharing of the emotion by the other and the feeling of the emotional state of the other and cognitive stage is the development of a mental representation of the emotional state. Compassion on the hand is a feeling of concern for another’s suffering combined with a motivation and an action to relieve the other’s suffering. Research has shown that there are distinct neural circuits for empathy and compassion - empathy was shown to activate the pathway involved with experiences of negative emotions and pain and compassion was shown to activate the pathway involved with positive affect and affiliation [3]. As such, it is important to delineate the terms used in literature as training in compassionate care will promote a pro-social state.

There is a stark absence of experimental studies testing interventions that increase compassionate behaviors and their impact on various outcomes such as patient benefit, provider benefit, healthcare system and payer benefit, and healthcare system cultural benefit [1]. Although other similar interventions to this study found significant changes in outcomes, many were complicated and impractical given their lengthy time requirement for implementation in hospitals and medical schools. For example, one study tested an intervention that included students participating in a once-a-week course; which included interviews with patients/physicians, supervised visits to the hospital, didactics and discussions of videotaped simulated consultations, over a four-month period [4]. Introducing longitudinal curricula such as this one may prove burdensome for already-crowded curricula at well-established medical schools.

Medical students and doctors experience high rates of psychological distress, including burnout, anxiety and depression [5, 6]. As such, there has been increasing interest in incorporating mindfulness-based interventions for student well-being. Based on improvements in psychological well-being and self-compassion, one narrative review concluded that mindfulness-based toolkits are indeed beneficial for medical students [7]. Mindfulness is the act of engaging in the non-judgmental awareness of the relevant aspects of an experience. It allows for moment-to-moment awareness while disengaging from “strong attachment to beliefs, thoughts, or emotions to experience emotional regulation and clarity in thinking [8]. As described, there is strong evidence to suggest that training medical students and physicians in compassion correlates with positive changes in at least one outcome measure, which can either be for the patient or the provider themselves [9]. Although this has been known for years, many medical schools have failed to implement compassion training for medical students, leading to the current “compassion crisis” we live in. While reasons for this failure have not been specifically delineated, it’s possible that traditional medical educators assumed compassion is inherently present in medical students. This assumption, though not completely deniable, is unlikely given the recent evidence that compassion is deficient in medical students [9]. Further research about compassionate care in medicine could demonstrate that compassion can be taught and developed in medical students without requiring burdensome training programs or seminars.

Currently, there is a gap in the literature for easily implementable, effective interventions to increase compassion in health care providers and students. This research aims to fill that gap by utilizing a mindfulness-focused approach to compassion to provide students with a “toolkit” to promote compassionate care.

## Methods

### IRB approval

This study was approved and granted a notice of exemption on 03/09/2022 by the Institutional Review Board at Stony Brook University (IRB2022-0119). Due to this exemption, informed consent did not need to be obtained for the study participants.

### Intervention

The intervention, the compassion selective, taught evidence-based cognitive exercises and practices followed by group discussions and written reflections based on thematic questions about those experiences. This selective was one option for a curricular requirement in the fourth year of medical school, amongst other courses described below. The intervention was designed in the following stepwise manner: understand what compassion means as a concept and as a practice via shared experiences, learn how to settle the mind through mindfulness-based practices, reflect on the emotions experienced by the self, reflect on the personal needs the self has and what corresponding behavior/activity do they engage, understand shared needs and values as a way to connect with others, learn what the barriers to connectedness are and how the stereotypes may influence our interactions with others, understand the concept of shared common humanity through mental exercises, and learn how to practice these on the go in a clinical setting.

The intervention included several exercises designed to cultivate compassion including: 1)asking the students to reflect in writing on common emotions and on the regulatory processes used to address those emotions; 2) reflecting in writing on the needs that underlie their behaviors; and 3) listening to another person’s story and reflecting on shared values, needs, and emotions.

The course was taught by a medical school faculty member from the Center for Medical Humanities, Compassionate Care and Bioethics and a 4th year medical student. The course was held over 40 h in 4 weeks − 16 h for lectures and small group discussions (2 h a day, 2 days a week for 4 weeks) and 24 h for self-study and assignments. The self-study included reading and analyzing papers related to the thematic topics being reviewed in class and reading chapters from The Art and Science of Compassion by Dr. Anges Wong [3]. The assignments included weekly self-reflections at the end of the week to discuss what was learned and how it may be implemented in their lives and an end-of-course project to create a presentation on an evidence-based article on compassion education.

### Study tools

The survey included demographic data, the Self-Compassion Scale short form, the Compassion Scale, and the Toronto Mindfulness Scale. The Self-Compassion Scale (26-item scale 5 point Likert scale; validated in 20 diverse samples *N* = 11,685 by Neff et al.) measures one’s ability for self-compassion [10]. The Compassion Scale (16-item 5-point Likert scale; validated in 20 diverse samples *N* = 11,685 by Neff et al.) entails the understanding of concepts and evaluates one’s ability to act with kindness, social connectedness, common humanity and mindfulness [11]. The Toronto Mindfulness Scale (13-item scale 5 point Likert scale; validated in one diverse sample *N* = 390 by Lau et al.) includes both the Curiosity subscale, which describes the ability to reflect with inquisitiveness, and the Decentering subscale, which focuses on the ability to be aware of one’s experience and distance one’s own emotions from it [12].

These scales were chosen as they are validated scales that consist of sub-scales that can examine separate components of self-compassion, compassion and mindfulness. The scales are theoretically coherent and allow brevity and flexibility of use in a classroom setting. The brief structure of the scales also allows for higher compliance and completion rates for the survey itself.

### Data collection

We anonymously surveyed medical students enrolled in the selective courses: Cultivating Compassion in Medicine, Addiction and Pain, Lifestyle Medicine, Telehealth Medicine, and Recognizing the Acutely-Ill Patient. The participants of these courses were fourth-year medical students enrolled to fulfill medical school requirements, and the courses ran from April 5th to April 28th, 2022. Students were surveyed using the online Qualtrics platform; pre- and post-course responses were collected over one week at the beginning of the course and one week at the end of the course. All the courses, including our selective, was taught at the Renaissance School of Medicine at Stony Brook University.

### Data analysis

For this project the Cultivating Compassion in Medicine course was our intervention, and students enrolled in courses that were not Cultivating Compassion in Medicine were designated as controls - Addiction and Pain, Lifestyle Medicine, Telehealth Medicine, and Recognizing the Acutely-Ill Patient. The results from the other courses, i.e. non-compassion selectives were pooled together for analysis. We conducted independent t-tests to assess potential associations between the pre-test responses for the validated scales between the groups (intervention and control), to determine whether there were any differences at baseline. We conducted independent t-tests to assess potential associations between the pre-test responses for the validated scales between the groups (intervention and control) to determine whether there were any differences at baseline. Since we had a small sample size we determined whether our variables followed a normal distribution in order to choose an appropriate statistical test. A Shapiro-Wilk test was performed and showed that the distribution of some of our variables departed significantly from normality. Therefore, we conducted Wilcoxon Signed Rank tests to compare paired samples and to assess potential associations within groups (intervention or control) between the pre- and post-survey responses for the validated scales and subscales that were utilized. Mann-Whitney U tests were used to compare non-paired samples, to determine whether there were any baseline differences between groups for the validated scales. The minimum criterion for significance was set at *p* < 0.05. Statistical analysis was performed using SPSS 28.0 statistical software (IBM Corporation).

**Table 1:** Participants in this study from each selective course

Course	Completed pre-survey	Completed post-survey
Cultivating Compassion in Medicine Selective	13	11
Addiction and Pain	11	3
Lifestyle Medicine	2	1
Telehealth Medicine	4	1
Recognizing the Acutely-Ill Patient	1	1

## Results

Participation in the study is described in Table 1. The 11 paired responses for Cultivating Compassion in Medicine were compared with the control group of the 6 paired responses for the other courses. The students were of varying demographic backgrounds, including age, ethnic group and gender. Before the start of the courses, no significant difference was found between the participants in the Cultivating Compassion in Medicine course and the pooled participants responses from the control courses for any of the four main measures: compassion (U = 67.5, *p* = 0.816), self-compassion (U = 68.5, *p* = 0.862), curiosity (U = 60.5, *p* = 0.522), or decentering (U = 62, *p* = 0.581). Tables 2, 3 and 4 demonstrates that students in the compassion course achieved a significant difference in self-compassion scores, compassion scores and the decentering component of the Toronto Mindfulness Scale.

**Table 2:** Mean scores and values demonstrating pre/post course differences for the compassion course and the control courses across the self-compassion and their subcomponents. Wilcoxon Signed Rank tests were performed to determine whether there was a significant increase in pre/post scores for the control and compassion groups

Scale	Control			Compassion		P value
	Mean pre-course score (SD)	Mean post-course score (SD)	P value	Mean pre-course score (SD)	Mean post-course score (SD)	
Self-Compassion (total)	32.8 (4.665)	34.2 (7.111)	0.461	31.7 (6.6)	40.5 (7.4)	0.008*
Self-Kindness	5.7 (0.8)	5.8 (1.0)	1	5.7 (1.6)	7.1 (1.5)	0.033*
Self-Judgment	6.2 (1.5)	6.3 (1.5)	0.564	6.6 (1.3)	5.0 (1.6)	0.037*
Common Humanity	4.8 (1.5)	5.3 (1.6)	0.102	4.6 (1.3)	7.0 (1.3)	0.003*
Isolation	8.3 (1.0)	7.0 (1.5)	0.109	7.5 (2.0)	6.2 (2.5)	0.028*
Mindfulness	7.8 (1.5)	7.0 (1.9)	0.180	7.3 (1.2)	8.0 (1.0)	0.074
Overidentification	7.0 (2.4)	6.7 (1.8)	1	7.8 (1.7)	6.5 (1.6)	0.007*

*Indicates a significant difference between pre/post-course scores (*p* < 0.05)

**Table 3:** Mean scores and values demonstrating pre/post course differences for the compassion course and the control courses across the compassion scale and their subcomponents. Wilcoxon Signed Rank tests were performed to determine whether there was a significant increase in pre/post scores for the control and compassion groups

Scale	Control			Compassion		P value
	Mean pre-course score (SD)	Mean post-course score (SD)	P value	Mean pre-course score (SD)	Mean post-course score (SD)	
Compassion (total)	61.5 (8.6)	56.7 (8.3)	0.144	62.7 (8.1)	68.7 (6.6)	0.012*
Kindness	16.0 (2.5)	14.5 (3.0)	0.194	16.6 (3.2)	18.4 (2.3)	0.024*
Common Humanity	13.7 (2.7)	13.3 (1.6)	1	14.5 (2.1)	15.9 (2.5)	0.063
Mindfulness	15.8 (3.2)	15.0 (2.4)	0.180	15.7 (2.2)	17.5 (1.8)	0.020*
Indifference	16.0 (3.0)	13.8 (3.7)	0.066	15.8 (2.4)	17.0 (2.6)	0.072

*Indicates a significant difference between pre/post course scores (*P* < 0.05)

### Self-compassion scale

The students in the compassion course achieved a significant (Z= -2.654, *p* = 0.008) increase in their total Self-Compassion score of 8.7 [95% CI 4.3 to 13.2] points. There was no statistically significant difference among the control course students (Z= -0.736, *p* = 0.461). The sub-components of this scale are shown in Table 2.

**Table 4:** Mean scores and values demonstrating pre/post course differences for the compassion course and the control courses across the Toronto Mindfulness Scale and their subcomponents. Wilcoxon Signed Rank tests were performed to determine whether there was a significant increase in pre/post scores for the control and compassion groups

Scale	Control			Compassion		P value
	Mean pre-course score (SD)	Mean post-course score (SD)	P value	Mean pre-course score (SD)	Mean post-course score (SD)	
Toronto Mindfulness Scale - Curiosity	17.8 (3.1)	16.7 (2.7)	0.785	18.0 (5.1)	22.4 (4.4)	0.012*
Toronto Mindfulness Scale - Decentering	16.0 (4.6)	17.3 (4.3)	0.357	16.5 (3.3)	19.5 (4.1)	0.113

*Indicates a significant difference between pre/post-course scores (*p* < 0.05)

### Compassion scale

The students in the compassion course achieved a significant (Z= -2.499, *p* = 0.012) increase in their total compassion score of 6.0 [95% CI 1.4 to 10.6] points. There was no statistically significant difference among the control course students (Z= -1.461, *p* = 0.144). The sub-components of this scale are shown in Table 3.

### Toronto mindfulness scale

The students in the compassion course achieved a significant (Z= -2.505, *p* = 0.012) increase in the curiosity component of the Toronto Mindfulness Scale of 4.4 [95% CI 1.0 to 7.7] points. There was no statistically significant difference in the decentering component of the scale (Z= -1.585, *p* = 0.113). There was no statistically significant difference among the control course students in either the curiosity component (Z= -0.272, *p* = 0.785) or the decentering component (Z= -0.921, *p* = 0.357). The sub-components of this scale are shown in Table 4.

## Discussion

Medical school is an environment in which many medical students and trainees experience intense stress, which, for some students, culminates into increased depression symptoms and suicidal ideation, as well as a low sense of personal accomplishment [5, 6]. Amidst all this, medical curricula demand students to practice self-care and compassionate care while providing little instruction on how to do so in practice [13]. Studies of medical trainees (residents and medical students) have found that mindfulness and meditation practices can influence a medical trainee’s levels of compassion, and many different methods have been employed to achieve this. An abridged mindfulness intervention to support wellness in first-year medical students showed a significant increase in self-compassion scores at the conclusion of the study and at six months [14]. Similarly, an 8-week mindfulness-based yoga intervention studying residents and medical students led to increased perceived compassion towards others post-intervention [15]. In this research, we set out to find if using a mindfulness-focused approach to compassion by providing medical students with a practical “toolkit” would, in fact, promote compassionate care, both for self and others. Throughout this curriculum, a combination of didactics in compassionate care and mindfulness theory and practical sessions to learn different methods of mindfulness and compassion helped students develop their personal toolkit to carry forward in their careers. The results convey a significant change in the ability of medical students who took the course to practice compassion. Self-compassion score pre-study showed no differences in the groups (compassion selective vs. other selectives), indicating no difference at baseline in self-compassion between groups before the intervention, while post-study scores were significantly higher in the compassion in medicine course group than the control group. Similarly, total compassion scores were not significantly different at baseline between the groups, and there was an increase in total compassion in this selective while no increase was seen in the control group. As groups do not differ at baseline, this would indicate that this difference is due to the selection, and not because of differences in population or self-selection into a course on compassion by people who might tend to be more compassionate. Students in the compassion course also significantly increased the curiosity component of the Toronto Mindfulness Scale. One of the major aspects of the compassion course focused on mindfulness-type interventions that students could employ. The proposed intervention provides a mindfulness-focused approach that provides a cognitive pathway to promoting self-compassion. The mental exercises and narratives they engage in help individuals identify their emotions, motivations and behaviors, allowing for appropriate emotional regulation. The intervention also provides strategies to enhance self-awareness, cognitive reappraisal and attentional control.

The finding in our study of an increased total compassion score for students who took the compassion course, which was not seen in the control group courses, indicates that the students who took the compassion course appeared to evidence growth in attentional stability, building mental representation of another’s emotions, understanding the shared human experience, connecting with another’s distress, and to be motivated to act to alleviate suffering. This supports our findings that mindfulness-based training courses can be effectively utilized to increase compassion among medical trainees [16, 17]. Our results demonstrate that a formal curriculum can aid in developing the “how” to be compassionate towards others by teaching emotional contextualization, perspective-taking and cognitive flexibility. Understanding the differences between empathy and compassion is also crucial to developing this cognitive pathway. Research from Paul Bloom states the effort involved in emotional empathy can emotionally exhaust individuals, erode relationships, and reduce our capacity to be concerned with others [18]. Instead, engaging in cognitive practices that allow reason to be present in the decision-making process for an altruistic act are better tools for the greater good [3]. The study also demonstrates that strategies using cognitive practices and constructive thinking can prime kindness, patience and prosocial attributes. Our intervention, which could be easily adapted to existing curricula that involve discussions of medical humanities and could even be taught remotely, educates students on techniques to help individuals identify personal needs and connect them to shared needs that exist in humanity.

Patients seek compassionate care; however, medical schools appear to struggle to teach future medical physicians how to nurture compassion [19]. We used certified scales to measure the effectiveness of the course on self-compassion, compassion and mindfulness. This increases the value of our intervention as a curricular tool. The framework taught in the course can be adapted to teach in a time-effective manner during multiple points of medical education.

As with all studies, there are limitations that should be noted when interpreting the results. The study found no baseline difference in compassion between the groups, but self-selection, personal motivation, and receptivity to training in compassion are confounding factors that should be addressed in a future study. Response bias should also be considered, as medical students may be pressured to answer differently due to the nature of the career they are entering into. Another limitation of the study is the small sample size. The intervention class enrolled fifteen students; however, only eleven completed the pre and post-surveys. The control group included multiple courses (sixty students taking their respective courses); however, only six completed the pre and post-surveys. While there is a large gap in study participation between groups, using a control group allowed us to address the bias of baseline differences in compassion levels due to self-motivation, as mentioned above. Our results indicate that utilizing mindfulness to foster compassion in medical students is a successful approach. Further studies focusing on our course need to be conducted with a larger sample size for additional insights into the course. A repeat post-survey course several months after the end of the course would also provide insight into the value of our course as a curricular tool.

## Conclusion

Proposed is a novel course designed to teach medical students how to practice compassion for themselves and their patients. The course was well received by early 4th-year medical students. It can be taught to medical students during any of the four years of schooling, albeit the recommendation is to expose the tools in the course early during medical education to provide sufficient time for incorporation and practice. The course can be used as an intervention and is customizable enough to apply to medical residents and physicians alike.

## Data Availability

All data is available in a secure server to use for further analysis or study. The datasets used and/or analyzed during the current study available from the corresponding author on reasonable request.
